# Dendritic cell expression of CD24 contributes to optimal priming of T lymphocytes in lymph nodes

**DOI:** 10.3389/fimmu.2023.1116749

**Published:** 2023-03-09

**Authors:** Xuejun Zhang, Chuan Yu, Jin-Qing Liu, Xue-Feng Bai

**Affiliations:** Department of Pathology and Comprehensive Cancer Center, The Ohio State University Medical Center, Columbus, OH, United States

**Keywords:** CD24, EAE (experimental autoimmune encephalitis), T lymphocytes, dendritic cells, T cell priming

## Abstract

CD24 is a GPI anchored cell surface glycoprotein whose function as a co-stimulatory molecule has been implicated. However, the function of CD24 on antigen presenting cells during T cell responses is not well understood. Here we show that in the CD24-deficient host, adoptively transferred CD4^+^ T cells undergo inefficient expansion and have accelerated cell death in lymph nodes, which results in insufficient priming of T cells. Insufficient expansion of T cells in the CD24-deficient host was not due to host anti-CD24 response by NK, T and B lymphocytes. Transgenic expression of CD24 on DC in CD24^-/-^ mice restored T cell accumulation and survival in draining lymph nodes. Consistent with these findings, MHC II tetramer staining also revealed that an antigen-specific polyclonal T cell response was reduced in lymph nodes of CD24^-/-^ mice. Taken together, we have revealed a novel role of CD24 on DC in optimal T cell priming in lymph nodes. These data suggest that CD24 blockade should lower unwanted T cell responses such as those in autoimmune diseases.

## 1 Introduction

CD24 is a glycosyl-phosphatidylinositol (GPI) anchored cell surface glycoprotein (1, 2). It is expressed on immature thymocytes, mature B lymphocytes and a variety of other types of cells such as dendritic cells (3–5) and regulatory T cells (6). CD24 disappears from T cells after maturation and has a rapid expression after T cell activation (7, 8). CD24 on T cells has been shown to be required for their homeostatic proliferation (9) and function (8). It has been reported that CD24 on antigen presenting cells (APCs) mediates CD28-independent co-stimulation of CD4 and CD8 T cells (3, 5, 10–14). Nevertheless, CD24 is not essential for the induction of immune responses to some antigens, as normal T cell and antibody responses were found in CD24-deficient mice (14–16). Moreover, CD24^-/-^ mice also had comparable parasite infection clearance rates (4). However, CD24 on dendritic cells has been shown to control the rapid homeostatic proliferation of syngeneic T cells in a lymphopenic model (17).

CD24 on immune cells has also been implicated in cell survival. CD24 transgenic expression in thymocytes (18) and pre-B cells (19) resulted in thymus atrophy and pre-B cell apoptosis. Cross-linking the murine CD24 induced apoptosis in B cell precursors (20) and thymocytes (21). We have previously shown that CD24 expression on thymic antigen presenting cells inhibits deletion of myelin antigen specific T lymphocytes (22). In addition, adoptively transferred myelin antigen specific T cells fail to survive and persist in CD24-deficient host (23). These results suggest that CD24 on antigen presenting cells such as DC may be related to the survival of lymphocytes.

Activation of T cells by DC in lymph nodes is a key initiating event in many immune responses including autoimmune responses. Experimental autoimmune encephalomyelitis (EAE) is an experimental model for human multiple sclerosis (MS). EAE is mediated by CD4^+^ T lymphocytes (24–27), which can be induced by immunization of mice with myelin antigens such as myelin oligodendrocyte glycoprotein (MOG) peptide 35-55. During the development of EAE and MS, myelin antigen specific T cells are activated in the lymph nodes and migrate into the central nervous system (CNS) where they execute their effector functions. We have previously shown that CD24 is required for the pathogenesis of EAE (23, 28), to understand the underlying mechanisms, in this study we have used the 2D2 TCR transgenic model to study a monoclonal MOG-specific T cell response in CD24^-/-^ mice. We found that in the CD24-deficient host, adoptively transferred myelin antigen specific T cells underwent inefficient expansion and accelerated cell death. Insufficient expansion of T cells in the CD24-deficient host was not due to host anti-CD24 response by NK, T and B lymphocytes. Transgenic expression of CD24 on DC in CD24^-/-^ mice restored T cell accumulation and survival in draining lymph nodes. Consistent with these findings, MHC II tetramer staining also revealed that an antigen-specific polyclonal T cell response was reduced in lymph nodes of CD24^-/-^ mice. Taken together, we have revealed a novel role of CD24 on DC in optimal T cell priming in lymph nodes.

## 2 Materials and methods

### 2.1 Mice

C57BL6, OT2 TCR transgenic mice were purchased from the Jackson Laboratory (Bar Harbor, ME). 2D2 TCR transgenic mice (29) were described before (22, 30). CD24^-/-^ mice in the C57BL6 background have been described (23, 28). C57BL6 mice-deficient for myelin antigen MOG (MOG^-/-^C57BL6) have been described before (31). 2D2^+^MOG^-/-^CD24^-/-^ and DC^CD24^CD24^-/-^

mice were described before (30). RAG-1^-/-^CD24^-/-^ mice were generated through breeding RAG-1^-/-^C57BL6 mice with CD24^-/-^ mice. All mice were bred and maintained in the animal facilities of The Ohio State University that are fully accredited by the American Association for Accreditation of Laboratory Animal Care.

### 2.2 Purification of CD4^+^ T cells from 2D2 or OT2 TCR transgenic mice and CFSE labeling

CD4^+^ T cells were purified from spleens and lymph nodes by negative selection. Briefly, spleen and lymph node cells from 2D2 or OT2 TCR transgenic mice were incubated with a cocktail of mAbs (anti-CD8 mAb TIB210, anti-FcR mAb 2.4G2 and anti-CD11c mAb N418). After removing the unbound antibodies, the cells were incubated with anti-IgG coated magnetic beads (Dynal Biotech). A magnet was used to remove the Ab-coated cells. The remaining cells were CD4^+^ T cells, typically with a purity of more than 90%. For CFSE labeling, purified 2D2 T cells were incubated with 1-2 μmol of CFSE in PBS at 37^0^C for 15 min and the labeling reaction was stopped by incubating cells with RPMI medium containing 10% FCS.

### 2.3 T cell adoptive transfer and immunization of mice

One to five million of purified CD4^+^ T cells from 2D2 or OT2 TCR transgenic mice were injected into each recipient mice (WT C57BL6, CD24^-/-^, RAG-1^-/-^CD24^-/-^, RAG-1^-/-^ and DC^CD24^CD24^-/-^ mice) i.v. Immediately after T cell injection, each recipient mouse was immunized with 100 μg MOG 35-55 (for mice receiving 2D2 T cells) or OVA 323-339 (for mice receiving OT2 T cells) emulsified in complete Freund’s adjuvant (CFA) s.c. At 17 h or 65 h after immunization, mice were sacrificed and cells from draining lymph nodes and spleens were analyzed for CFSE dilution and apoptosis (Annexin V staining) as we described before (32, 33).

### 2.4 Antibodies and flow cytometry

The following antibodies were used in the experiments according to the manufacturer’s recommendations: FITC-, PE-, PerCp-, or APC-labeled anti-CD3 (17A2, 100206, Biolegend), -CD4 (RM4-5, 100516, Biolegend), -CD8α (53-6.7, 2002714, eBioscience), -CD11c (N418, 117310, Biolegend), -CD24 (M1/69, 12-0242-83, eBioscience), -CD45 (30-F11, 103108, Biolegend), -Vα3.2 (RR3-16, 553219, BD Biosciences), -Vβ5.1/5.2 (MR9-4, 553190, BD Biosciences), -Vβ11 (RR3-15, 553198, BD Biosciences), -B220 (RA3-6B2, 103212, Biolegend) and -F4/80 (BM8, 123108, Biolegend). For flow cytometry analysis, cells were incubated with antibodies (with a final dilution of 1:200) on ice for 30 min followed by extensive washing. Cells were then analyzed on a FACScalibur cytometer (Becton Dickinson, Mountain View, CA, USA). Data were analyzed using the flowjo software (Tree Star, Inc., OR).

### 2.5 NK cell depletion

To deplete NK cells before T cell adoptive transfer, 300 μg of anti-NK1.1 antibody (PK136, BioXcell) was injected into each WT and CD24^-/-^ mouse i.p. Two days later each mouse received 3 x 10^6^ of CFSE-labeled 2D2 T cells i.v. followed by immunization with MOG 35-55/CFA. Efficacy of NK cell depletion was examined by labeling cells with CD45, CD3 and NK1.1 followed by flow cytometry.

### 2.6 Induction and assessment of EAE

For induction of EAE in RAG-1^-/-^ and RAG-1^-/-^CD24^-/-^ mice, mice of 8 to12 weeks of age first received purified 2D2 T cells (2 x 10^6^/mouse) i.v. Twenty four hours later, 2D2 T cell recipients were immunized s.c. with 200 µg MOG 35-55 in CFA (containing 400 µg of Mycobacterium tuberculosis) in a total volume of 100 µL. Mice received 100 ng of pertussis toxin (List Biological, Campbell, California, USA) in 200 µL PBS in the tail vein immediately after the immunization and again 48 hours later. The mice were observed every day and were scored on a scale of 0 to 5 with gradations of 0.5 for intermediate scores: 0, no clinical signs; 1, loss of tail tone; 2, wobbly gait; 3, hind limb paralysis; 4, moribund; and 5, death.

#### 2.6.1 T cell proliferation assay

CD4^+^ T cells were purified from day-10 draining lymph node cells from MOG-peptide immunized WT and CD24^-/-^ mice. A total of 5 × 10^5^ cells per well were stimulated with indicated concentrations of MOG peptide in the presence 5 × 10^5^ cells per well of irradiated (20 Gy) syngeneic splenocytes for 60 hours. The cultures were pulsed with [^3^H]thymidine (1 μCi/well; ICN Pharmaceuticals Inc., Costa Mesa, CA) for another 12 hours, and incorporation of [^3^H]thymidine was measured in a liquid scintillation β-plate counter.

#### 2.6.2 Tetramer staining

APC labeled MOG_38–49_-IA^b^ and APC-labeled GP_66–77_-IA^b^ tetramers were provided by the National Institute of Allergy and Infectious Diseases Tetramer Core Facility at Emory University. Day-10 draining lymph node cells were incubated with 4 µg/ml MOG_38–49_-IA^b^ (2 h) or GP_66–77_-IA^b^ tetramers (2 h) in complete Braff’s medium at 37°C. Cells were also stained for CD4 and CD8 before flow cytometry analysis was performed.

### 2.7 Statistical analysis

Data are presented as the mean ± SD. Normal distribution was assumed for all data. N values for each experiment are indicated in figure legends. Unpaired or paired, two-tailed student’s t test was used to calculate P values. A P value less than 0.05 was used to define statistical significance.

## 3 Results

### 3.1 CD24 is required for optimal expansion of T cells in lymph nodes

**Figure 1:**
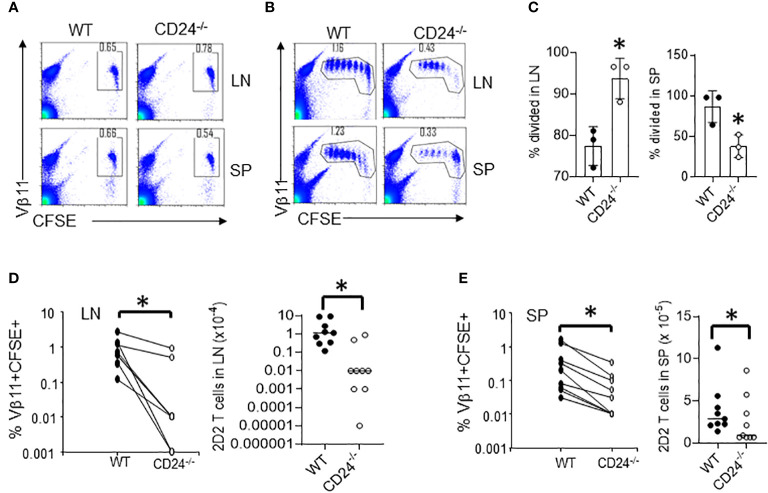
Proliferation of 2D2 T cells in CD24-deficient mice. CFSE-labeled 2D2 T cells (5 x 10^6^/mouse) were injected into WT or CD24^-/-^ mice intravenously (i.v.) followed by subcutaneous (s.c.) injection of MOG 35-55 emulsified in CFA. **(A, B)**. Percentages of 2D2 T cells (CFSE^+^Vβ11^+^) in draining lymph nodes and spleens of WT and CD24^-/-^ mice at 17 h **(A)** and 65 h **(B)** after immunization were analyzed by flow cytometry. Data shown in A-B are representative of eight experiments with similar results. **(C)** Percent of divided 2D2 T cells in lymph nodes and spleens at 65 h. Representative data from one experiment are shown. **(D, E)**. Percentages and total numbers of 2D2 T cells in draining lymph nodes **(C)** and spleens **(D)** of WT and CD24^-/-^ hosts at 65 h were quantified. Data shown in **(C, D)** were pooled from three independent experiments performed in sex and age-matched mice. SP, spleen; LN, draining lymph nodes. *p<0.05, student’s t test was used for the comparison.

**Figure 2:**
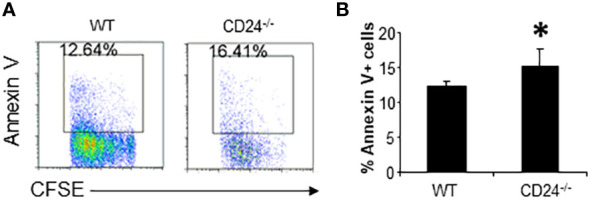
2D2 T cell survival in lymph nodes of CD24^-/-^ and WT mice. **(A)**. Flow cytometry analysis of 2D2 T cell death in draining lymph nodes of WT and CD24^-/-^ mice. Data shown are gated on Vβ11^+^CFSE^+^ cells. 5 x 10^6^ CFSE-labeled 2D2 T cells were injected into WT or CD24^-/-^ mice i.v. followed by immunization with MOG 35-55/CFA. **(B)**. Percent of Annexin V^+^ 2D2 T cells in the draining lymph nodes of WT and CD24^-/-^ mice. Five mice per group were used for the experiment. *p<0.05, student’s t test was used for the comparison. Data represents 8 experiments with similar results.

We have previously shown that CD24^-/-^ mice are relatively resistant to the induction of EAE. To determine the role of CD24 in the activation and expansion of myelin antigen specific T cells, we purified CD4^+^ T cells from 2D2 TCR transgenic mice, whose TCR recognizes MOG35-55, and labeled purified 2D2 T cells with CFSE. 1-5 x 10^6^ CFSE-labeled 2D2 T cells were then injected into each WT or CD24^-/-^ mouse, followed by immunization of the recipient mice with MOG peptide/CFA subcutaneously (s.c.). At different times after immunization, cell division and accumulation in draining lymph nodes and spleens were analyzed by flow cytometry. As shown in **Figure 1A**, similar percentage numbers of 2D2 T cells were detected in the draining lymph nodes and spleens of WT and CD24^-/-^ mice by 17 h, and T cells remained undivided. By 65 h, T cells divided up to 7 times in the draining lymph nodes and spleens of WT and CD24^-/-^ mice (**Figure 1B**). Intriguingly, although we found that more 2D2 T cells divided in lymph nodes of CD24^-/-^ mice than in WT mice, their percentage numbers were lower (**Figure 1B**); additionally, spleens of CD24^-/-^ mice also contained less divided cells compared to WT mice (**Figure 1C**). As a result, the percentages, and total numbers of 2D2 T cells were significantly reduced in both draining lymph nodes and spleens of CD24^-/-^ mice compared to WT mice (**Figures 1D, E**). Since 2D2 T cells underwent similar divisions in the draining lymph nodes of CD24^-/-^ mice, we considered the less efficient expansion of 2D2 T cells could be due to increased cell death. As demonstrated in **Figure 2A** and quantified in **Figure 2B**, we found that higher percentages of 2D2 T cells underwent apoptosis in the draining lymph nodes of CD24^-/-^ mice than in WT mice, as reflected by the percentage of 2D2 T cells which stained positive for Annexin V.

**Figure 3:**
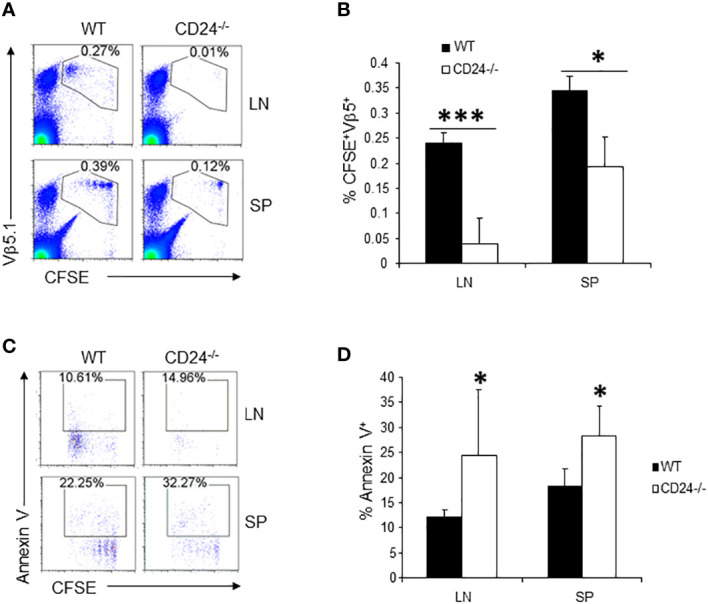
Proliferation and survival of OT2 T cells in CD24^-/-^ mice. **(A, B)**. 1 x 10^6^ CFSE-labeled OT2 T cells were injected into WT or CD24^-/-^ mice i.v. Percent of OT2 T cells (CFSE^+^Vβ5.1^+^) in draining lymph nodes and spleens were quantified at 65 h after immunization with OVA 323-339/CFA. Five mice per group were used in this experiment. Data shown are Mean ± SD. *p<0.05, ***P<0.001; student’s t test was used for the comparison. **(C, D).** Percent of 2D2 T cells that underwent apoptosis in draining lymph nodes and spleens of WT and CD24^-/-^ mice at 65 h after immunization with OVA 323-339/CFA. Five mice per group were used in this experiment. Data shown are Mean ± SD. *p<0.05, student’s t test. Data shown represent two experiments with similar results.

To determine if the observations made on 2D2 T cells also apply to T cells specific to other antigens, we labeled OT2 T cells with CFSE and injected 1 x 10^6^ CFSE-labeled OT2 T cells into OVA peptide immunized CD24^-/-^ and WT mice. As demonstrated in **Figure 3**, by 65 h, the majority of the OT2 T cells underwent multiple divisions in the draining lymph nodes and spleens of WT mice. However, T cells were almost undetectable in the draining lymph nodes in CD24^-/-^ mice and had much lower percentage numbers in the spleens of CD24^-/-^ mice (**Figures 3A, B**). In the draining lymph nodes and spleens of CD24^-/-^ mice, higher percentages of OT2 T cells underwent apoptosis (**Figures 3C, D**).

### 3.2 Insufficient expansion of 2D2 T cells in the lymph nodes of CD24^-/-^ mice was not due to host response to CD24

**Figure 4:**
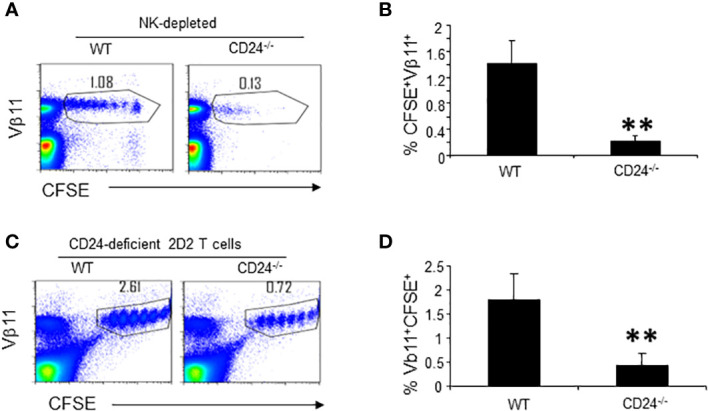
Host response to CD24 is not responsible for insufficient expansion of T cells in lymph nodes of CD24^-/-^ mice. **(A, B).** Depletion of NK cells does not rescue 2D2 T cells in the lymphoid organs of CD24^-/-^ mice. 300 μg of PK136 antibody (anti-NK1.1) was injected into each WT and CD24^-/-^ mouse i.p. Two days later each mouse received 3 x 10^6^ of CFSE-labeled 2D2 T cells i.v. followed by immunization with MOG 35-55/CFA. Flow cytometry analysis of lymph node cells was performed at 65 h. Three mice per group were used in this experiment. Data shown are Mean ± SD. **p<0.01, student’s t test. Data are representative of two experiments with similar results. **(C, D)**. 5 x 10^6^ of CFSE-labeled CD24-deficient 2D2 T cells (obtained from 2D2^+^CD24^-/-^MOG^-/-^ mice) were injected into each WT and CD24^-/-^ mice i.v. followed by immunization with MOG 35-55/CFA. Flow cytometry analysis of lymph node cells was performed at 65 h. Five mice per group were used in this experiment. Data shown are Mean ± SD. **p<0.01, student’s t test. Data are representative of two experiments with similar results.

The insufficient expansion of 2D2 T cells in the CD24^-/-^ mice could be due to host responses to CD24, as activated T cells rapidly gain CD24 expression (7, 8). Since insufficient expansion occurs within 72 h, host innate cells such as NK cells may mediate depletion of activated T cells. To test this possibility, we treated WT and CD24^-/-^ mice with a high dose of anti-NK1.1 antibody intraperitoneally (i.p.), followed by i.v. injection of CFSE-labeled 2D2 T cells and immunization with MOG35-55/CFA 48 h later. As shown in **Figure S1**, anti-NK1.1 treatment nearly completely depleted NK cells in treated mice. However, NK depletion did not restore T cell accumulation in draining lymph nodes of CD24^-/-^ mice (**Figures 4A, B**). Thus, it is unlikely that host NK cells mediated deletion of the adoptively transferred T cells in CD24^-/-^ mice.

To test if insufficient expansion of T cells in the draining lymph nodes of CD24^-/-^ mice was due to host anti-CD24 response, we labeled 2D2 T cells from 2D2^+^CD24^-/-^MOG^-/-^ mice with CFSE and injected 5 x 10^6^ CFSE-labeled CD24-deficient 2D2 T cells into each WT or CD24^-/-^ mouse i.v. followed by MOG35-55/CFA immunization. As demonstrated in **Figures 4C, D**, in CD24^-/-^ mice, CD24-deficient 2D2 T cells still underwent insufficient expansion. Thus, the insufficient expansion of 2D2 T cells in the CD24^-/-^ mice were not due to host responses to CD24.

**Figure 5:**
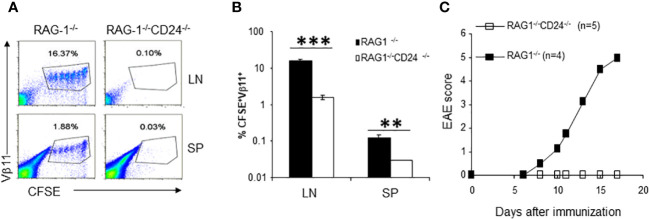
2D2 T cell proliferation and pathogenicity in RAG-1^-/-^CD24^-/-^ mice. **(A, B)**. 2D2 T cells accumulated in the draining lymph nodes [upper panel of **(A)**] and spleens [lower panel of **(A)**] of RAG-1^-/-^ mice but not in RAG-1^-/-^CD24^-/-^ mice. 5 x 10^6^ CFSE-labeled 2D2 T cells were injected into each recipient mouse i.v. followed by immunization with MOG 35-55/CFA. Flow cytometry analysis of lymph node cells were performed at 65 h. Percent of CFSE^+^Vβ11^+^ cells in each group were plotted in **(B)**. **P<0.01, ***P<0.001 by student's t test. Five mice per group were used in this experiment and data shown represents Mean ± SD. Student’s t test was used for the statistical analysis. **(C)**. RAG-1^-/-^CD24^-/-^ mice receiving 2D2 T cells failed to develop EAE. 2 x 10^6^ of CD4 T cells from 2D2 TCR transgenic mice were injected into each RAG-1^-/-^ and RAG-1^-/-^CD24^-/-^ mouse i.v. The recipients were then immunized for EAE using MOG35-55/CFA/PT. Data shown represents two experiments with similar results.

To test the possibility that T and B lymphocytes caused depletion of 2D2 T cells in the peripheral lymphoid organs of CD24^-/-^ mice, we injected CFSE-labeled 2D2 T cells into RAG-1^-/-^CD24^-/-^ and RAG-1^-/-^ mice followed by MOG35-55 immunization. As shown in **Figures 5A, B**, we also observed dramatically reduced 2D2 T cells in the draining lymph nodes of RAG-1^-/-^CD24^-/-^ mice. To test the possibility that 2D2 T cells might preferentially migrate into the CNS and cause CNS inflammation, we adoptively transferred 2 x 10^6^ 2D2 T cells into each RAG-1^-/-^CD24^-/-^ and RAG-1^-/-^ mouse followed by immunization with MOG35-55/CFA and 100 ng of pertussis toxin. As demonstrated in **Figure 5C**, while all RAG-1^-/-^ mice developed lethal EAE, no RAG-1^-/-^CD24^-/-^ mice developed disease. Thus, 2D2 T cell death, but not their preferential migration to the CNS was responsible for the disappearance of 2D2 T cells in the CD24^-/-^ host.

### 3.3 Dendritic cell expression of CD24 is sufficient for expansion and survival of T cells in lymph nodes

**Figure 6:**
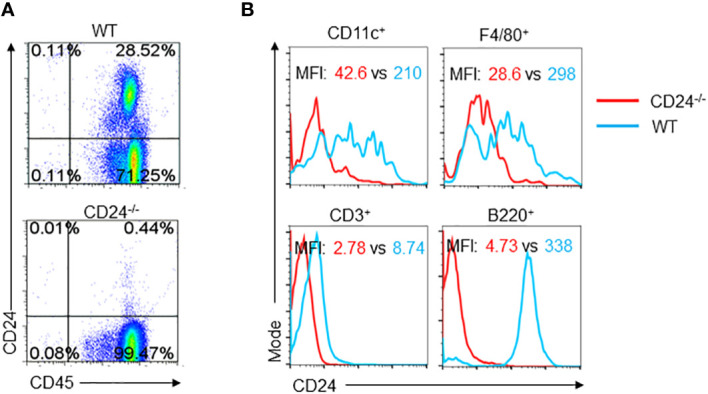
Expression of CD24 in lymph node cells. **(A)**. Lymph node cells from WT and CD24^-/-^ mice were stained for CD45 and CD24. **(B)**. CD24 expression on different subsets of CD45^+^ Cells. Lymph node cells from WT and CD24^-/-^ mice were stained for CD45, CD24 and one of the subset markers. Data represent five experiments with similar results.

To understand the requirement of CD24 expression during T cell response in lymph nodes, we examined lymph node cells for the expression of CD24. As shown in **Figure 6A**, expression of CD24 is mainly found in the CD45^+^ compartment of cells. Among the CD45^+^ cells, B cells have the highest expression of CD24; CD11c^+^ DC have similar levels of CD24 expression to macrophages (F4/80^+^); CD3^+^ T cells had low levels of expression (**Figure 6B**).

**Figure 7:**
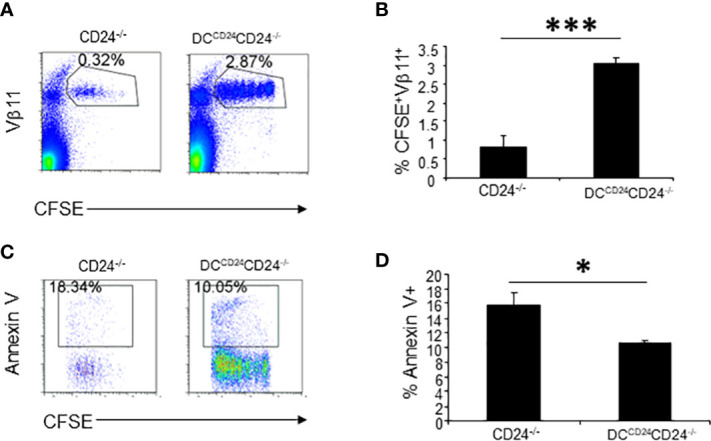
CD24 expression on DC contributes to proliferation and survival of 2D2 T cells. 5 x 10^6^ of CFSE-labeled 2D2 T cells were injected into DC^CD24^CD24^-/-^ or CD24^-/-^ mice i.v. followed by immunization with MOG 35-55 emulsified in CFA. Representative staining **(A)** and quantification of % CFSE^+^Vβ11^+^ cells **(B)** are shown. Five mice per group were used in this experiment and data shown are Mean ± SD. ***P < 0.001. Student’s t test was used for the statistical analysis. **(C, D)**. Apoptosis of 2D2 T cells was quantified at 65 h after immunization with MOG 35-55/CFA. Five mice per group were used for this experiment. Data shown are Mean ± SD. Student’s t test was used for the statistical analysis. Data represent two experiments with similar results. *P < 0.05.

Since CD24 expression on T and B cells are not necessary for optimal proliferation of T cells (**Figure 5**), we hypothesized that CD24 on DC was necessary for optimal expansion of T cells. To test if CD24 expression on DC is required for optimal proliferation of 2D2 T cells, DC^CD24TG^ mice were crossed with CD24^-/-^ mice for two generations and we generated mice with CD24 expression only on DC (DC^CD24^CD24^-/-^ mice) (30). In these mice, all CD11c^+^ cells uniformly expressed CD24 (**Figure S2**). CFSE-labeled 2D2 T cells were injected into CD24^-/-^ and DC^CD24^CD24^-/-^ mice followed by immunization with MOG35-55/CFA. As demonstrated in **Figure 7A** and quantified in **Figure 7B**, restoration of CD24 expression on DC resulted in the restoration of T cell proliferation and accumulation in the draining lymph nodes. Percentages of apoptotic 2D2 T cells was also significantly reduced in the lymphoid nodes of DC^CD24^CD24^-/-^ mice compared to CD24^-/-^ mice (**Figures 7C, D**). Thus, restoration of CD24 expression on DC resulted in optimal proliferation of 2D2 T cells in draining lymph nodes.

### 3.4 Reduced autoantigen-specific CD4^+^ T cell priming in lymph nodes of CD24^-/-^ mice

**Figure 8:**
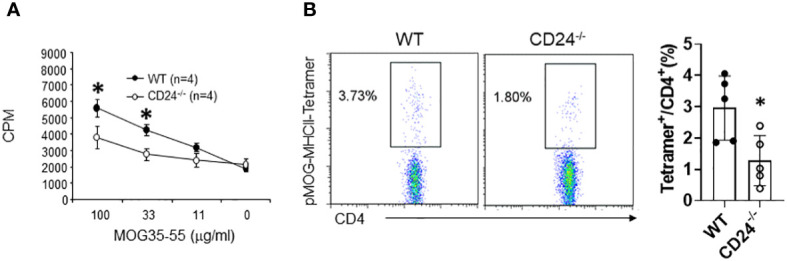
Reduced priming of MOG peptide-specific T cells in lymph nodes from CD24^-/-^ mice. WT and CD24^-/-^ mice were immunization with MOG 35-55/CFA. On day-10 after immunization, lymph node CD4^+^ T cells were assessed for proliferation **(A)** and pMOG-MHCII-tetramer staining **(B)**. Data shown represents two **(A)** and three **(B)** experiments with similar results. *P<0.05 by student’s t test.

We previously analyzed autoantigen MOG-specific T cell responses in draining lymph nodes of MOG peptide immunized CD24^-/-^ and WT mice using a global lymph node cell proliferation assay and ELISPOT assay (28). In that study, we detected a mall reduction of MOG-reactive T cell proliferation in lymph nodes of CD24^-/-^ mice, but no reduction of MOG peptide-reactive T cell response by ELISPOT assay. To determine if CD24-deficiency affect the priming of polyclonal CD4^+^ T cells, we immunized WT and CD24^-/-^ mice with MOG35-43/CFA. On day 10 after immunization, we purified CD4^+^ T cells from lymph nodes and assessed their proliferative response to MOG peptide. As shown in **Figure 8A**, CD4^+^ T cells in lymph nodes from CD24^-/-^ mice showed reduced proliferation in response to MOG peptide compared to CD4^+^ T cells in lymph nodes from WT mice. Additionally, MOG peptide-specific tetramer staining also revealed reduced frequencies of MOG-specific T cells in draining lymph nodes from CD24^-/-^ mice (**Figure 8B**).

## 4 Discussion

In this study, we have found that CD24 expression on DC is required for optimal priming of T cells in lymph nodes. This observation is supported by the following lines of evidence. First, in the CD24-deficient lymph nodes, T cells underwent inefficient expansion in response to antigens; second, the inefficient expansion of T cells in lymph nodes were not due to inefficient T cell homing to lymph nodes or due to host anti-CD24 response and third, restoration of CD24 on DC can correct T cell priming defects in lymph nodes. Consistent with these observations, we also find that CD24 contributed to optimal priming of an autoantigen-specific T cells in lymph nodes. A few previous studies have revealed the importance of CD24 on DC in T cell responses. We have previously shown that CD24 on DC is required for thymic generation of autoantigen-specific T cells (30). Li et al. have shown that CD24 on DC is critical for the control of homeostatic proliferation of autoreactive T cells (17). Kim et al. have shown that CD24^+^ DCs in lymph nodes promote the differentiation of viral antigen-specific T cells into effector T cells (34). Thus, our current study differs from all previous studies and presents a novel finding that CD24 in lymph node DCs is required for the expansion and survival of antigen-specific T cells.

In the CD24-deficient host, adoptively transferred myelin antigen specific T cells underwent inefficient expansion and accelerated cell death. Inefficient expansion of T cells in lymph nodes were not due to inefficient homing of T cells to lymph nodes, as comparable numbers of T cells were detected at 17 h after T cell transfer (**Figure 1A**). Inefficient expansion of T cells in the draining lymph node was not likely due to inefficient division of T cells when seeing antigen in lymph nodes since all T cells (2D2 and OT2) divided similar or more times in draining lymph nodes. The resulting lower T cell numbers were not due to migration of 2D2 T cells into peripheral tissues, since we also detected reduced numbers of T cells in the spleens of CD24^-/-^ mice (**Figures 1E**, **3A, B**). In addition, in RAG-1^-/-^CD24^-/-^ mice that received 2D2 T cells, T cells were barely detected in the CNS (not shown) and no EAE developed in those mice. Thus, it is likely that CD24 in lymph nodes is required for the survival of activated T cells. Indeed, we have found that in draining lymph nodes and spleens of CD24^-/-^ mice, antigen specific T cells underwent more apoptosis (**Figure 2**, **Figures 3C, D**). Previous studies have revealed that CD24 on immune cells is involved in cell survival. CD24 transgenic expression in thymocytes (18) and pre-B cells (19) resulted in thymus atrophy and pre-B cell apoptosis. Cross-linking the murine CD24 induced apoptosis in B cell precursors (20) and thymocytes (21). This study is the first to show that CD24 in lymph nodes is required for the expansion and survival of activated T cells.

The less efficient expansion and disappearance of adoptively transferred T cells in immune lymph nodes suggests that the host might have mounted anti-CD24 immune response thereby depleting CD24^+^ T cells. However, based on three lines of evidence, this possibility can be ruled out. First, depletion of NK cells did not rescue T cell expansion in the lymph nodes of CD24^-/-^ mice. Second, adoptive transfer of T cells into RAG-1^-/-^CD24^-/-^ mice, also failed to restore T cell expansion in lymph nodes. Finally, adoptively transferred CD24-deficient T cells also poorly expanded in lymph nodes of CD24^-/-^ mice. Thus, the most likely explanation for the poor expansion of T cells in CD24-deficient lymph nodes was that host antigen presenting cells such as DC, need CD24 expression to maintain an optimal T cell response.

The experiments performed in RAG-1^-/-^CD24^-/-^ mice suggest that CD24 on host T and B lymphocytes is not required for optimal expansion of T cells in lymph nodes. In contrast, in DC^CD24^CD24^-/-^ mice T cell proliferation and survival was restored (**Figure 7**). Although our data do not rule out the roles of CD24 expressing macrophages, our data do suggest that restoration of CD24 in DC is sufficient. Thus, we have identified a new role of CD24 on DC in optimal T cell priming in lymph nodes. At this stage, we do not know which subset of DC in lymph nodes are most important in optimal priming of T cells in draining lymph nodes. However, Kim et al. have reported (34) the importance of two CD24^+^ migratory DC subsets in T cell activation and differentiation. They found that CD103^+^ migratory DC expressed higher CD24 and promoted T cell differentiation into effector T cells while CD11b^hi^ migratory DC stimulated T cells to obtain central memory phenotype and increased T cell retention in draining lymph nodes. Thus, it is likely that CD24 expression on both subsets are needed for optimal priming of T cells.

Since CD24-deficiency does not appear to affect the expression of CD80, CD86 and MHC II molecules in lymph node DCs (**Figure S3**), the lack of optimal priming of T cells in draining lymph nodes cannot be explained by these traditional antigen presentation and co-stimulation mechanisms. CD24 is known to interact with CD24 itself (35) and P-selectin (36). However, since we observed that T cells deficient for CD24 do not show defects in proliferation and survival in WT mice (**Figure 4C**), CD24 homotypic interaction between DC and T cells is not responsible for lack of optimal proliferation. Additionally, a tri-molecule model of how CD24 works has been proposed (37, 38), in particular, CD24-HMGB1 on DC has been shown to engage RAGE on T cells to promote T cell priming (34). It is thus highly likely that these CD24 partners are involved in optimal priming of T cells in lymph nodes. Finally, it is known that CD24-deficient DCs produce more inflammatory cytokines such as IL-6, MCP-1, and TNF-α and restoration of CD24 in DCs inhibited the production of these cytokines (38). Thus, increased production of inflammatory cytokines and other factors in the absence of CD24 in DC can also be a factor affecting proliferation and survival of T cells in draining lymph nodes (39). Overall, we propose that during a T cell activation in lymph nodes, CD24 on DC provides critical signals either directly or indirectly to T cells and inhibits activation-induced T cell death.

In this work, we also revealed that induction of polyclonal autoantigen MOG-specific T cell responses is impaired in draining lymph nodes of MOG peptide immunized CD24^-/-^ mice (**Figure 8**). This finding is different from our previous observation that T cell responses were largely normal in lymph nodes of CD24^-/-^ mice (28). We consider the difference was caused by using different methodologies. Previously we used a global lymph node cell proliferation assay and ELISPOT assay to evaluate lymph node T cell response. ELISPOT assay likely detects both low affinity and high affinity T cells, while MHC II-tetramer is known to detect high affinity T cells leaving low affinity T cells undetected (40).

Although we have revealed a critical function of CD24 on DC for T cell optimal priming in lymph nodes, some previous studies have demonstrated that CD24 is not required for T cell responses (14, 15). Moreover, CD24^-/-^ mice also had comparable parasite infection clearance rates (4). The discrepancy of these observations could be due to the different settings used. It is also possible that some antigen (such as systemic antigen) specific T cell responses do not entirely rely on interactions in lymph nodes. For instance, L-selectin has been shown to be critical for naïve T cell entry into lymph nodes and essential for some T cell responses (41, 42). Under certain situations, such as high antigen dose or stronger priming conditions (43), T cell responses can also be primed in spleen of L-selectin deficient mice. Nonetheless, lymph nodes have been shown to be essential for many types of immune responses.

Taken together, in this work we have identified a novel role of CD24 on DC in optimal priming of antigen-specific T cells. This finding together with our previous findings that CD24 is required for thymic generation of autoantigen specific T cells (22, 30) and expansion of autoreactive T cells in target tissues (17, 23) suggest that CD24 blockade should lower unwanted T cell responses such as those in autoimmune diseases.

## Data availability statement

The original contributions presented in the study are included in the article/**Supplementary Material**. Further inquiries can be directed to the corresponding author.

## Ethics statement

The animal study was reviewed and approved by IACUC of The Ohio State University.

## Author contributions

XZ performed most experiments and summarized data. CY performed cell proliferation and pMHCII-tetramer staining experiments. J-QL develop mouse strains for the experiments. X-FB designed all experiments and performed some experiments, wrote the manuscript and generated funding support. All authors contributed to the article and approved the submitted version.
