# Supplementary material for: Dendritic cell expression of CD24 contributes to optimal priming of T lymphocytes in lymph nodes
Source: Front Immunol. 2023 Mar 9;14:1116749. doi: 10.3389/fimmu.2023.1116749 (PMC10033833; doi:10.3389/fimmu.2023.1116749)
Supplement: Supplementary file 1 [file DataSheet_1.pdf]

Supplemental Figures and Figure legends

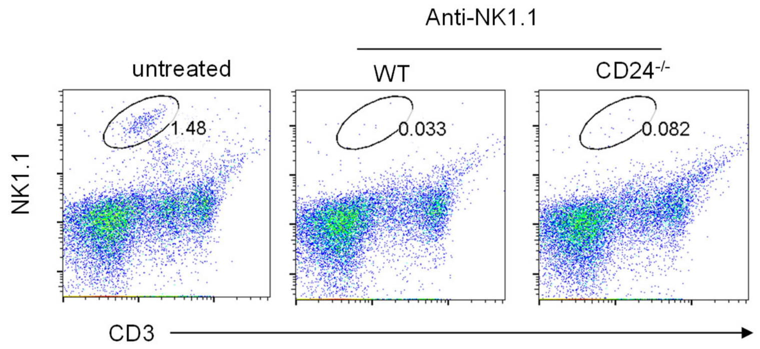

Fig.S1. Efficacy of NK depletion. 300 µg of PK136 antibody (anti-NK1.1) was injected into each WT and CD24$^{-/-}$ mouse i.p. Flow cytometric analysis of NK cell depletion was performed on day 5. Data shown are representative of mice from each group.

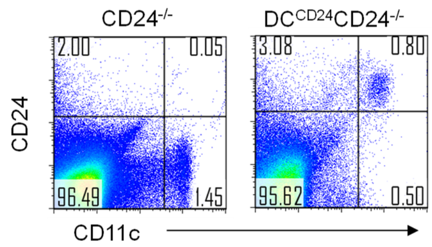

Fig.S2. Uniformed expression of CD24 in DCs from DC^{CD24}CD24$^{-/-}$ mice. Flow cytometry was used to analyze CD24 expression in DCs. Data shown are representative of mice from each group.

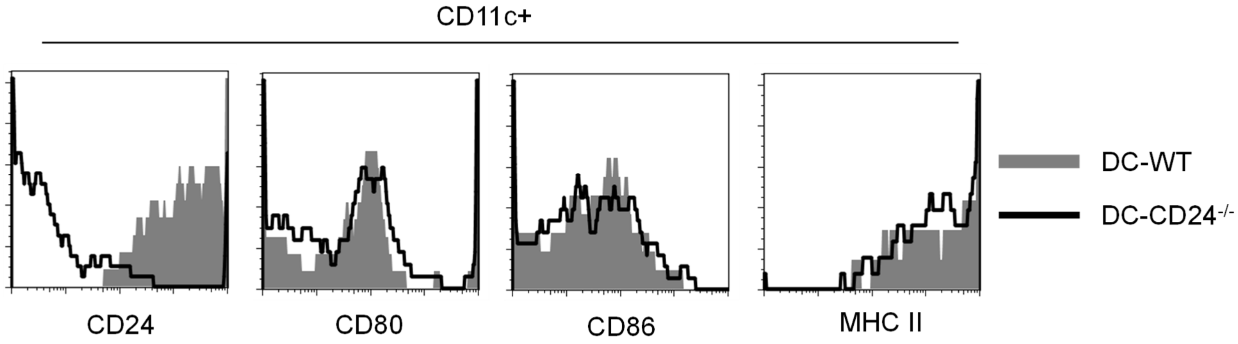

Fig.S3. Expression of MHC II and co-stimulatory molecules in DC from CD24$^{-/-}$ mice. Flow cytometry was used to analyze DCs in immune lymph nodes. Data shown are from representative mouse from each group.
